# Comprehensive overview of the quality of plant‐ And animal‐sourced proteins based on the digestible indispensable amino acid score

**DOI:** 10.1002/fsn3.1809

**Published:** 2020-08-25

**Authors:** Laure Herreman, Paul Nommensen, Bart Pennings, Marc C. Laus

**Affiliations:** ^1^ Avebe Innovation Center Groningen The Netherlands

**Keywords:** DIAAS, digestibility, essential amino acids, sustainability, vegetable protein

## Abstract

Indispensable amino acid (IAA) composition and standardized ileal digestibility (SID) of five animal‐ and 12 plant‐based proteins were used to calculate their respective Digestible Indispensable Amino Score (DIAAS) according to the three age categories defined by the Food and Agriculture Organization (FAO). Mean IAA content and mean SID obtained from each protein dataset were subsequently used to simulate optimal nutritional quality of protein mixtures. Datasets revealed considerable variation in DIAAS within the same protein source and among different protein sources. Among the selected protein sources, and based on the 0.5‐ to 3‐year‐old reference pattern, pork meat, casein, egg, and potato proteins are classified as excellent quality proteins with an average DIAAS above 100. Whey and soy proteins are classified as high‐quality protein with an average DIAAS ≥75. Gelatin, rapeseed, lupin, canola, corn, hemp, fava bean, oat, pea, and rice proteins are classified in the no quality claim category (DIAAS <75). Potato, soy, and pea proteins can complement a broad range of plant proteins, leading to higher DIAAS when supplied in the form of protein mixtures and at specific ratios. Such complementarity highlights the potential to achieve an optimal nutritional efficiency with plant proteins alone.

## Graphical abstract

Indispensable amino acid (IAA) content and ileal digestibility coefficients of 17 protein sources were used to determine the Digestible Amino Acid Score (DIAAS). Considerable DIAAS variations within the same protein source and between protein sources were obtained. The differences observed in the protein quality lead to opportunities to enhance their nutritional efficiency in the form of protein mixtures at specific ratios. This overview also highlights the considerations needed to compose a reliable DIAAS database.

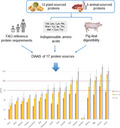

## 1 INTRODUCTION

New protein sources have emerged in recent years to support the transition toward more sustainable food production dedicated to human nutrition. Animal‐sourced proteins, for example, from milk and meat, have shown to have a substantial impact on greenhouse gases and are known to contribute to depletion of natural sources (Aiking, 2014; Godfray et al., 2018). These proteins are included to a large extent in Western diets, and their consumption is gradually increasing in developing countries (Godfray et al., 2018). While it has been recommended to shift global protein consumption toward plant‐based proteins (Boland et al., 2013; Pyett, Vet, Trindade, Zanten, & Fresco, 2019), attention should be paid to the nutritional quality of new and alternative protein sources.

Dietary protein quality is primarily characterized by their indispensable amino acid (IAA) content. IAAs cannot be synthetized by the human body and must be obtained from the diet. The current standard to evaluate the nutritional quality of proteins is the Protein Digestibility Corrected Amino Acid Score (PDCAAS). Due to several drawbacks of this method (Sarwar, 1997), the Food and Agriculture Organization (FAO) recommends another procedure called Digestible Indispensable Amino Acid Score (DIAAS) (FAO, 2013). DIAAS addresses the limitations of the PDCAAS method by considering the ileal digestibility of individual amino acids (AAs), with the growing pig as preferred model over the rat, and by avoiding truncation of the score obtained. Furthermore, reactive digestible lysine rather than total digestible lysine should be considered to determine the DIAAS of processed and cooked foods. The FAO DIAAS report also recommends classification of proteins using quality categories based on the DIAAS value: <75 (no quality claim); 75–99 (high‐quality protein); and ≥100 (excellent quality protein). The FAO has updated the age‐related AA reference scoring patterns and recommends that the general population be categorized according to three distinct age‐related reference patterns: 0–6 months (infant), 0.5–3 years (children), and > 3 years (rest of the population).

Recent studies compared various protein sources based on their AA composition (Gorissen et al., 2018; Sá, Moreno, & Carciofi, 2020; van Vliet, Burd, & van Loon, 2015). However, these data do not inform to which extent a specific protein can be digested to meet the human body requirements. The most extensive studies to date provide DIAAS values of eight protein sources (Cervantes‐Pahm, Liu, & Stein, 2014; Mathai, Liu, & Stein, 2017). To broaden the comparison of DIAAS to more protein sources, IAA profiles and IAA standardized ileal digestibility (SID) data were used to calculate the DIAAS values of 17 protein sources according to the three reference scoring patterns.

Standardized ileal digestibility is preferred over apparent ileal digestibility (AID) since the latter does not correct for the AA endogenous loss inherent to the body function (Stein, Sève, Fuller, Moughan, & de Lange, 2007). Endogenous AAs are secreted into the small intestine, and an estimated 20%–35% is not reabsorbed by the body before reaching the distal ileum (de Lange, Sauer, Mosenthin, & Souffrant, 1989; Souffrant et al., 1993). Applying AID as digestibility coefficient therefore leads to underestimation of the actual AA digested. We also combine protein sources to optimize their nutrition profile, for which protein digestibility coefficients must be additive. SID therefore provides a more accurate and appropriate measurement of the AA ileal digestibility of single protein sources or protein mixtures (Stein, Pedersen, Wirt, & Bohlke, 2005). To improve comparison between results, studies involving rodents, gilts, sows, piglets, weanling pigs, and finishing pigs were excluded and instead only data obtained with growing pigs were used. In addition, only protein material containing at least 10% crude protein, and falling in categories such as seeds, meals, flours, concentrates, and isolates, was included in the investigation. Material in the forms of hulls, brans, or peels was excluded, being less suitable sources of protein for human consumption. The obtained datasets were subsequently used to simulate protein mixtures and highlight the complementarity of proteins at specific ratios to obtain higher values than that of the individual proteins.

## 2 MATERIALS AND METHODS

### 2.1 Data collection from the literature

Five animal (whey, casein, egg, gelatin, and pork meat) and 12 plant protein sources (soy, pea, lupin, fava bean, rapeseed, canola, hemp, wheat, potato, oat, rice, and corn) were selected, based on their occurrence in the Western diet or by the growing interest in the vegetarian, vegan, or flexitarian markets. Growing pig intervention studies providing complete IAA composition, crude protein content (CP), and IAA standardized ileal digestibility (SID) were selected.

### 2.2 DIAAS of a single protein source

The most limiting digestible indispensable amino acid content (DIAA) defines the DIAAS value of a protein. DIAA ratios were determined according to the three reference pattern scores defined by FAO: infant (0–6 months), children (0.5–3 years), and children older than 3 years, adolescents, and adults (FAO, 2013).

For a given IAA “y,” DIAA ratio is calculated as follows:

(1) $$\text{DIAAy ratio}=\frac{\text{IAAy}\times \text{SIDy}}{\text{Reference pattern score IAAy}}$$

where IAA y is expressed as mg/g CP.

The lowest DIAA ratio leads to the DIAAS value of a protein:

(2) DIAAS=100×lowest DIAA ratio among IAAs

Mean DIAAS values were obtained from the lowest mean DIAA for each protein source. Graphs were generated with Microsoft Excel Office 365 and Minitab software version 18.1.

### 2.3 DIAAS of protein mixtures

Digestible indispensable amino acid content of protein mixtures were calculated as detailed by FAO and according to the reference pattern score of 0.5‐ to 3‐year‐old population group (FAO, 2013). Average values of single proteins for IAAy and SIDy were used to estimate the DIAAys of protein mixtures. The latter is written as a linear combination with respect to the mixing ratios Ri. For a mixture of protein 1 (P_1_), protein 2 (P_2_) up to protein *n* (P_n_), this results in:

(3) $$\text{DIAAy}=c_{\text{y,1}}\times R_{1}+c_{\text{y,2}}\times R_{2}+\cdots +c_{\text{y,n}}\times R_{n}$$

where:

(4) $$c_{y,i}=\frac{\text{IAAyi}\times \text{SIDyi}}{\text{Reference pattern score IAAy}}$$

and

(5) $$R_{i}=\frac{\text{Mi}}{\sum \text{Mi}}$$

Note that Equation (4) is independent of material crude protein content. Mi is the amount of pure protein from protein source i. By using pure protein, DIAAy remains independent of the protein content of individual material used in the mixture.

For a mixture of two protein sources, Equation (3) can be rewritten by applying R_2_ = 1 ‐ R_1_:

(6) $$\text{DIAAy}=\left(c_{\text{y,1}}‐c_{\text{y,2}}\right)\times R_{1}+c_{\text{y,2}}$$

The optimal mixture provides the maximum DIAAS among all possible ratios. The corresponding R_1_ value was determined with the Solver tool available in Microsoft Excel, using the constraint 0 ≤ R_1_ ≤ 1. The objective was set to find the highest DIAAS ≤ 100.

For a mixture of three protein sources, a similar scheme was used:

(7) $$\text{DIAAy}=c_{\text{y,1}}\times R_{1}+c_{\text{y,2}}\times R_{2}+c_{\text{y,3}}\times R_{3}$$

And using R_3_ = 1 ‐ R_1_ ‐ R_2_, this results in:

(8) $$\text{DIAAy}=\left(c_{\text{y,1}}‐c_{\text{y,3}}\right)\times R_{1}+\left(c_{\text{y,2}}‐c_{\text{y,3}}\right)\times R_{2}+c_{\text{y,3}}$$

The values of R_1_, R_2_, and R_3_ for the optimal mixture were also calculated with the Solver tool available in Microsoft Excel. Equation (8) was optimized by modifying both R_1_ and R_2_. Values of R_1_ and R_2_ were constrained to 0 ≤ R_1_ ≤ 1 and 0 ≤ R_2_ ≤ 1 with the objective to find the highest DIAAS ≤ 100.

Similar to Equation (2), the DIAAS score is obtained from the lowest DIAA value of the protein mixture:

(9) $$\text{DIAAS}\;(P_{1}+P_{2}+\ldots +P_{n})=100\times \text{lowest DIAA}\;(P_{1}+P_{2}+\ldots +P_{n})$$

## 3 RESULTS

### 3.1 DIAAS variation within protein datasets

Studies providing complete IAA composition, CP content, and IAA SID were selected (Table 1). The amount of data available from the literature varies according to the protein source. Digestibility of corn, soy, canola, and wheat proteins has been extensively studied in growing pigs, evidenced by the relatively large number of datasets obtained through numerous references (Table 1 and Table S1). Contrarily, complete data on ileal digestibility of amino acids from hemp and casein are limited to 1 and 2 datasets, respectively.

**TABLE 1:** Datasets collected to calculate DIAAS of protein sources

Protein source	Number of datasets^a^	References
Wheat	37	Cervantes‐Pahm et al., (2014); CVB, (2016); Lee, Ahn, Son, & Kim, (2019); Mathai et al., (2017); McGhee & Stein, (2018); NRC, (2012); Pedersen et al., (2007); Sauvant et al., (2004); Wang, Osho, & Adeola, (2018); Woyengo et al., (2014); Zhao et al., (2019)
Rice	3	CVB, (2016); Gottlob et al., (2006)
Oats	8	Abelilla, Liu, & Stein, (2017); Cervantes‐Pahm et al., (2014); CVB, (2016); NRC, (2012); Sauvant et al., (2004)
Rapeseed	31	CVB, (2016); Huang et al., (2018); Hulshof et al., (2016); Li et al., (2015); Maison & Stein, (2014); Sauvant et al., (2004)
Pea	22	CVB, (2016); Grosjean et al., (2000); Mathai et al., (2017); NRC, (2012); Sauvant et al., (2004); Stein & Bohlke, (2007)
Soy	43	Baker & Stein, (2009); Berrocoso et al., (2015); Cervantes‐Pahm & Stein, (2008); CVB, (2016); Hulshof et al., (2016); Kong, Kang, Kim, & Kim, (2014); Lee et al., (2019); Mathai et al., (2017); NRC, (2012); Sauvant et al., (2004); Son, Park, Park, & Kim, (2019)
Whey	12	CVB, (2016); Gottlob et al., (2006); Mathai et al., (2017); NRC, (2012); Sauvant et al., (2004)
Casein	2	CVB, (2016); NRC, (2012)
Egg	3	Woyengo et al., (2015); Zhang et al., (2015)
Canola	26	Berrocoso et al., (2015); Liu et al., (2016); Liu, Song, Maison, & Stein, (2014); Maison & Stein, (2014); NRC, (2012); Park, Ragland, Helmbrecht, Htoo, & Adeola, (2019); Seneviratne et al., (2010); Son et al., (2019); Wang et al., (2018); Xue, Ragland, & Adeola, (2014)
Corn	44	Almeida, Petersen, & Stein, (2011); CVB, (2016); Ji, Zuo, Wang, Li, & Lai, (2012); Lee et al., (2019); NRC, (2012); Sauvant et al., (2004); Son et al., (2019); Xue et al., (2014); Zhang et al., (2019)
Potato	5	Beelen G.M., (1999); CVB, (2016); NRC, (2012); Sauvant et al., (2004)
Hemp	1	Presto, Lyberg, & Lindberg, (2011)
Gelatin	3	NRC, (2012); Petersen, Smiricky‐Tjardes, & Stein, (2005)
Lupin	4	CVB, (2016); Lee et al., (2019); NRC, (2012)
Fava bean	3	NRC, (2012); Sauvant et al., (2004)
Pork	9	Bailey & Stein, (2019)

^a^ Each dataset combines complete amino acid composition of the protein (% CP) and standardized ileal digestibility of the nine indispensable amino acids (including cysteine and tyrosine).

**FIGURE 1:**
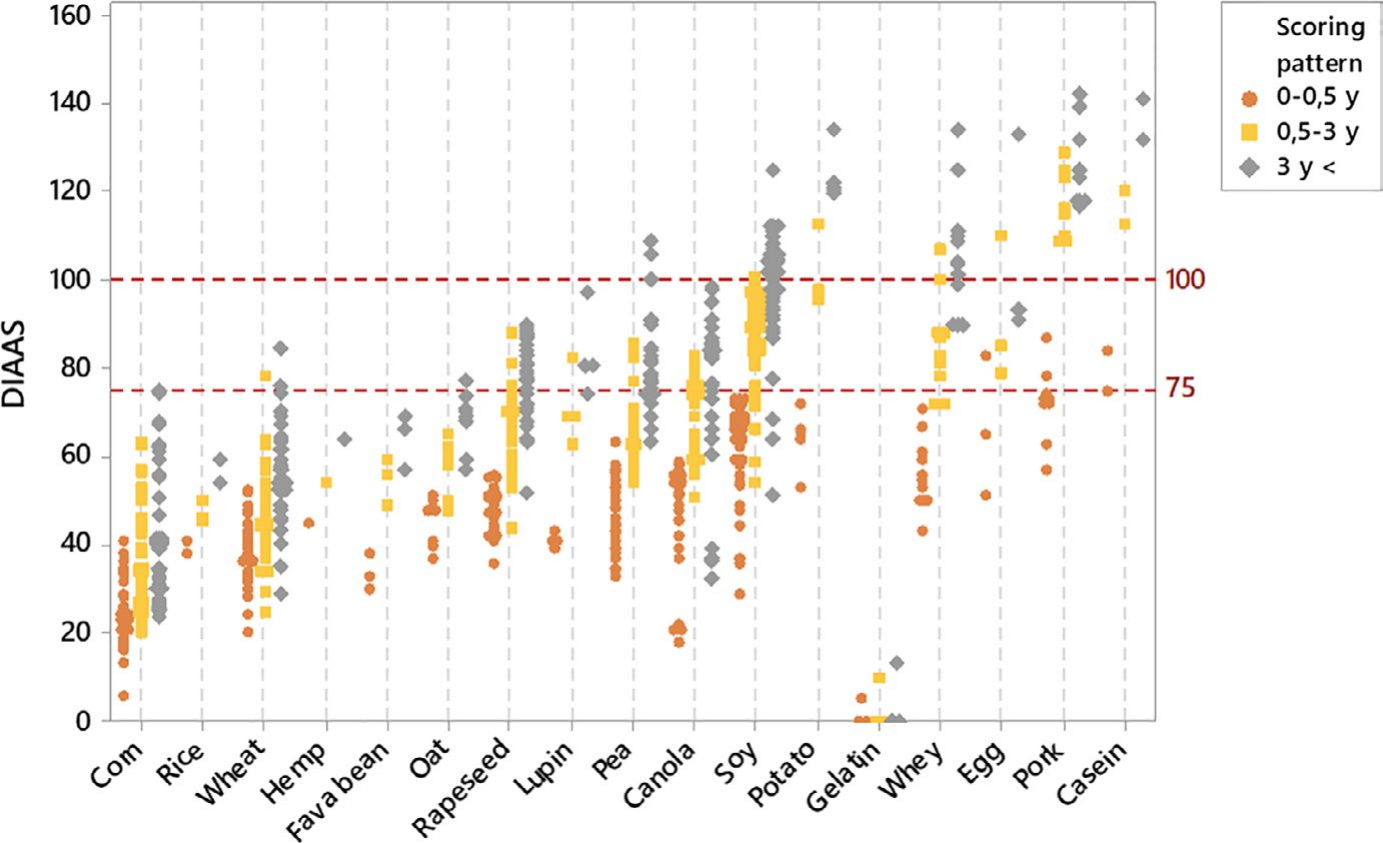
Variation in DIAAS obtained from SID and IAA data available from pig intervention studies. DIAAS calculated for each scoring pattern as defined by FAO (2013): infant (0–0.5 years), children (0.5–3 years), and children older than 3 years, adolescents, and adults

Each retrieved dataset was used to obtain the DIAAS value according to the three scoring patterns. As displayed in Figure 1, DIAAS values are scattered across the different protein quality categories for many protein sources. Based on the 0.5‐ to 3‐year‐old scoring pattern, soy and wheat proteins possess DIAAS values in all three quality categories (<75, no quality protein claim; 75–99, high‐quality protein claim; and ≥100, excellent quality protein claim), while scores of wheat, rapeseed, lupin, pea, potato egg, and canola proteins fall in two of the quality categories. This is further highlighted with the broad absolute DIAAS variations observed within most protein datasets. Based on the 0.5‐ to 3‐year‐old scoring pattern, DIAAS values of wheat, rapeseed, and corn proteins display the greatest disparity with respective DIAAS ranges of 53, 43, and 44. DIAAS variation tends to decrease for the infant group (0–0.5 years old), with range values of 32, 20, and 34 for wheat, rapeseed, and corn proteins, respectively. Such decreases in variation can be explained by higher IAA requirement expressed in the scoring pattern and the shift in limiting amino acid as detailed in Table S2.

Given the varying protein content among protein sources, the absence of measurements of antinutritional factors (ANFs), and the limited description of the process used to obtain the various protein sources, a selection cannot be based on a specific determinant. For this reason, the average DIAAS value obtained from each protein dataset was selected.

### 3.2 DIAAS per protein source

Mean DIAAS values obtained from the lowest average DIAA reveal large differences among plant‐derived proteins, varying from 36 ± 14.9 (corn) to 100 ± 7.3 (potato), and among animal‐derived proteins, varying from 2 ± 3.0 (gelatin) to 117 ± 11.7 (casein), considering the scores obtained for 0.5‐ to 3‐year‐old group (Table 2). For this age group, the limiting amino acid of protein obtained from cereal grains—such as corn, wheat, hemp, rice, canola, oat, rapeseed—is lysine (Lys), while the leguminous sources of protein (fava bean, pea, lupin, soy) are limited by the sulfur‐containing amino acids methionine and cysteine (Met + Cys). Potato protein, which interestingly shows a high DIAAS value, is derived from a tuberous plant and therefore does not belong to any of these categories (Table 2).

**TABLE 2:** Digestible indispensable amino acid scores of various protein sources according to the 0.5‐to 3‐year‐old reference pattern score

Protein source	Histidine	Isoleucine	Leucine	Lysine	Met + Cys	Phe + Tyr	Threonine	Tryptophan	Valine	DIAAS	Limiting AA^a^
Corn	110 ± 29.7	90 ± 14.6	162 ± 58.2	36 ± 14.9	126 ± 22.2	140 ± 42.8	86 ± 10.2	52 ± 35.4	90 ± 14.4	36	Lys
Rice	93 ± 7.0	89 ± 17.4	80 ± 12.4	47 ± 2.3	104 ± 11.0	119 ± 29.6	75 ± 4.1	114 ± 28.6	95 ± 18.0	47	Lys
Wheat	118 ± 21.7	91 ± 10.5	87 ± 11.1	48 ± 10.6	127 ± 19.4	109 ± 16.9	78 ± 7.1	127 ± 17.8	92 ± 9.8	48	Lys
Hemp^b^	124 ± NA	106 ± NA	85 ± NA	54 ± NA	121 ± NA	131 ± NA	87 ± NA	‐	99 ± NA	54	Lys
Fava bean	108 ± 4.1	106 ± 2.2	95 ± 5.4	95 ± 4.3	55 ± 5.1	119 ± 3.4	91 ± 6.2	68 ± 7.8	83 ± 2.2	55	Met + Cys
Oat	91 ± 11.4	100 ± 4.2	94 ± 4.9	57 ± 5.8	151 ± 52.9	135 ± 9.2	85 ± 5.9	110 ± 17.2	102 ± 3.4	57	Lys
Rapeseed	107 ± 8.0	90 ± 4.9	78 ± 5.0	67 ± 10.3	125 ± 14.3	92 ± 12.3	97 ± 6.5	106 ± 9.4	92 ± 4.6	67	Lys
Lupin	121 ± 16.1	104 ± 27.2	89 ± 19.3	75 ± 12.3	68 ± 12.7	121 ± 35.6	97 ± 22.7	72 ± 22.5	78 ± 14.6	68	Met + Cys
Pea	99 ± 9.7	101 ± 13.1	87 ± 11.5	110 ± 10.8	70 ± 12.3	116 ± 16.3	94 ± 7.9	77 ± 7.1	83 ± 9.8	70	Met + Cys
Canola	105 ± 6.9	93 ± 9.9	79 ± 7.8	72 ± 9.2	121 ± 10.4	97 ± 6.1	97 ± 12.2	112 ± 19.5	87 ± 9.1	72	Lys
Soy	119 ± 9.4	124 ± 8.3	102 ± 6.1	96 ± 9.0	91 ± 11.5	147 ± 8.3	105 ± 6.0	132 ± 21.1	95 ± 7.3	91	Met + Cys
Potato	100 ± 7.3	156 ± 9.2	143 ± 11.2	122 ± 4.6	115 ± 6.0	210 ± 18.2	165 ± 12.0	128 ± 13.7	138 ± 5.1	100	NA
Gelatin	34 ± 9.5	34 ± 10.6	35 ± 8.7	60 ± 11.5	27 ± 10.3	36 ± 13.0	46 ± 4.9	2 ± 3.0	46 ± 8.6	2	Trp
Whey	85 ± 10.8	166 ± 23.2	138 ± 22.9	131 ± 25.2	132 ± 21.6	101 ± 14.0	174 ± 22.8	180 ± 47.0	116 ± 14.3	85	His
Egg	101 ± 11.7	129 ± 25.5	103 ± 16.2	133 ± 58.4	123 ± 53.2	144 ± 18.9	106 ± 14.1	129 ± 49.7	105 ± 32.3	101	NA
Casein	147 ± 9.4	153 ± 4.3	141 ± 6.6	134 ± 4.3	117 ± 5.0	201 ± 8.0	130 ± 4.3	159 ± 13.4	148 ± 2.7	117	NA
Pork	197 ± 13.6	153 ± 11.1	122 ± 9.2	157 ± 10.7	128 ± 10.7	148 ± 10.4	145 ± 10.1	144 ± 17.1	117 ± 9.0	117	NA

Note

Data expressed as mean of individual DIAA values ± standard deviation.

^a^ Limiting AA when DIAAS <100, NA not applicable when DIAAS ≥100.

^b^ Dataset does not include tryptophan measurements. This is however the only publication reporting the ileal digestibility of hemp in pig studies. These data were therefore included for comparison.

**FIGURE 2:**
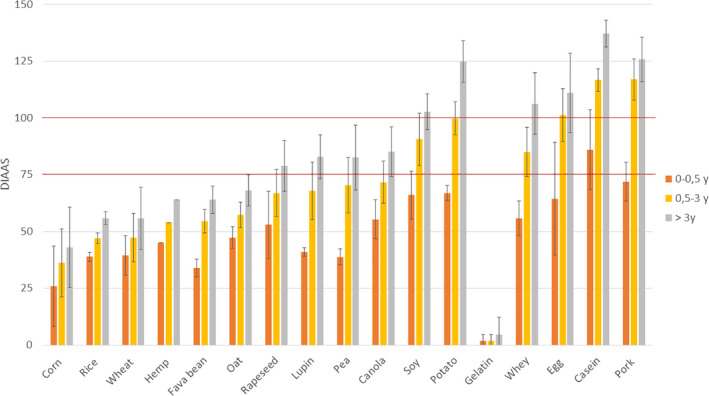
Average DIAAS of various protein sources according to the three reference pattern scores: infant (0–0.5 years), children (0.5–3 years), and children older than 3 years, adolescents, and adults. Error bars represent standard deviation

Due to different protein requirements between age groups, the limiting amino acid of the protein sources varies per age group. Tryptophan (Trp), lysine (Lys), and phenylalanine and tyrosine (Phe + Tyr) are the most common limiting IAAs for infants (Table S2). In this population group, maximum scores are obtained for casein (86 ± 17.5), followed by pork meat (72 ± 8.6) and potato protein (67 ± 3.3) (Figure 2). The most limiting IAAs for children, adolescents, and adults (>3 years old) are Lys, Met + Cys, and histidine (His) for cereal, leguminous, and potato proteins, respectively (Table S3).

A protein source reaching a DIAAS of 100 or above indicates that none of its amino acids is limiting and this sole protein source should be able to meet physiological requirements. Among the proteins selected in this study, potato protein, egg protein, casein, and pork meat reach this level based on the 0.5‐ to 3‐year‐old reference pattern. For a protein source with a DIAAS lower than 100, different strategies are possible to ensure an adequate protein intake based on such protein. A first option is to increase the protein intake of the limiting protein until the physiological requirement is reached. Higher protein intake has indeed shown to increase amino acid uptake in plasma (Gorissen et al., 2016). For example, based on the selected datasets, less than three portions of corn protein (2.8*36 = 101) would theoretically be needed to meet these requirements while 1.45 portions of pea protein (1.45*70 = 101) would be required. A second option is to combine protein sources and ensure complementarity of their amino acids to reach a higher DIAAS.

### 3.3 Toward a higher DIAAS with protein combinations

**FIGURE 3:**
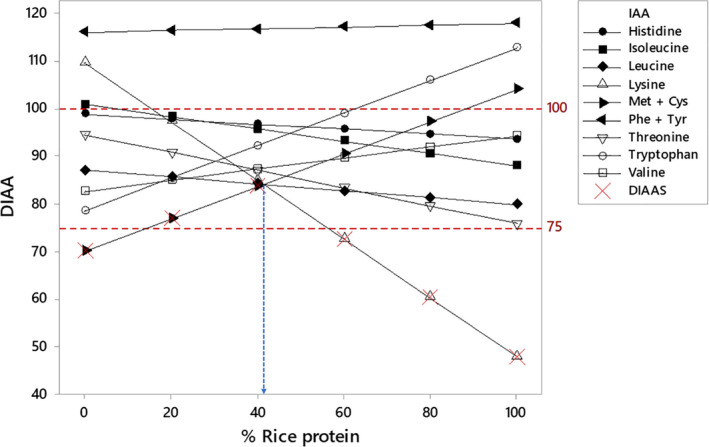
Variation in digestible indispensable amino acid values and resulting DIAAS in pea/rice protein mixture. Illustration based on the average SID and average IAA composition obtained from pea protein and rice protein datasets. A maximum DIAAS of 84 can be obtained when 41% rice protein is composing the pea/rice protein mixture as indicated by the blue arrow

The digestibility score of each indispensable amino acid highlights the potential of complementarity between protein sources (Table 2). Cereal‐based proteins, scoring low in Lys but high in Met + Cys, can to some extent complement leguminous proteins, scoring high in Lys but low in Met + Cys. Potato protein, which scores higher than 100 for each DIAA value, can also serve to increase DIAAS of many proteins. The degree of complementarity depends on the ratio of the combined protein sources, as illustrated in Figure 3.

Multiple simulations were used to calculate the maximum DIAAS of mixtures according to Equations (6), (8), and (9), enabling mixtures such as oat/lupin, fava bean/corn, and pea/wheat to reach the high‐quality protein range (Table 3). Not all leguminous/cereal protein combinations will lead to higher scores: If Lys is the second or third limiting IAA in leguminous protein, the DIAAS increase will be minimal. This is the case of lupin protein scoring 75 for Lys (Table 2). Among these mixtures, potato protein shows the ability to increase the DIAAS of most plant protein sources to 100. In combination with casein, egg protein, or pork meat, most plant proteins can reach a DIAAS of 100 until a certain ratio is reached (Table S4).

**TABLE 3:** Improved DIAAS as a result of optimal plant protein combination

Plant protein mixture	Max. DIAAS^a^ (≤100)	Ratio
Oat/lupin	76	7/93
Oat/lupin/soy	91	10/10/80
Oat/lupin/potato	100	10/20/60
Fava bean/corn	64	75/25
Fava bean/corn/soy	85	10/20/70
Fava bean/corn/potato	100	15/20/65
Fava bean/rapeseed	82	55/45
Pea/wheat	85	60/40
Pea/wheat/soy	90	25/20/55
Pea/wheat/potato	100	25/25/50
Canola/pea	84	35/65
Canola/pea/soy	92	25/15/60
Canola/pea/potato	100	35/35/30
Soy/canola	92	85/15
Soy/wheat	90	90/10
Soy/wheat/potato	100	25/20/55
Soy/oat	92	90/10
Corn/potato	100	25/75
Corn/soy	88	15/85
Wheat/potato	100	30/70
Lupin/potato	100	30/70

^a^ DIAAS value derived from average IAA content and average SID per protein sources and calculated according to Equations (6), (8), and (9). Based on 0.5‐ to 3‐year‐old reference pattern score.

## 4 DISCUSSION

### 4.1 Factors influencing DIAAS variability

Protein IAA content and SID coefficients were used to determine DIAAS values for 17 protein sources. Broad absolute DIAAS variations can be observed within most protein datasets. This can be attributed to the type of material fed to the growing pigs.

Genotype or cultivar considerably influences AA content, AA composition, and SID (Spindler et al., 2016; Strang, Eklund, Rosenfelder‐Kuon, Htoo, & Mosenthin, 2017; Zhao et al., 2019). Similarly, the content of antinutritional factors (ANFs), for example, glycoalkaloid, glucosinolates, protease inhibitors, phytate, and saponins, differs among genotypes (Oomah et al., 2011; Sharma, Kaur, Goyal, & Gill, 2014). Along with processing conditions, ANFs are well‐known to influence plant protein digestibility (Sá, Moreno, & Carciofi, 2019; Sarwar Gilani, Wu Xiao, & Cockell, 2012). For instance, Luo et al. (Luo & Xie, 2013) reported that phytate and trypsin inhibitor contents increase with dehulling of fava beans, but was most effectively reduced by first soaking the beans before dehulling followed by an autoclaving step. Pastuszewska et al. (Pastuszewska, Tuśnio, Taciak, & Mazurczyk, 2009) also highlighted the variations in the activity of solanidine glycoalkaloids and trypsin inhibitors in potato protein concentrate among process conditions from various potato starch production sites. Furthermore, fermentation or enzymatic treatments of soybean have also shown their potential to reduce the content of ANFs leading to an increased AA digestibility in young pigs (Zhang et al., 2013). In the studies selected for this analysis, the diverse processes used to obtain the protein materials—such as specific heat treatment, solvent extraction conditions, or enzymatic reaction—are not consistently described; the resulting activity of ANFs is rarely investigated. Moreover, many of the datasets used in the current study are based on raw feed ingredients. Such material often displays a higher ANF content and ANF activity than dietary protein used in food products. Feedstuffs in their raw state do not reflect the processed and cooked forms of food used for human consumption. Processing and especially cooking can considerably affect amino acid composition and digestibility of proteins, resulting in a different DIAAS (Bailey, Mathai, Berg, & Stein, 2019; Friedman, Gumbmann, & Masters, 1984). While heat treatment can inactivate some of the ANFs, it can also induce molecular alterations making the protein more resistant to the action of digestive proteases or on the contrary more accessible (Carbonaro, Cappelloni, Nicoli, Lucarini, & Carnovale, 1997; Duodu, Taylor, Belton, & Hamaker, 2003; Liu, Zheng, & Chen, 2019). This is also illustrated in the present study in which pea protein obtains an average DIAAS of 70 while after extrusion its DIAAS can increase to 82 or 86 depending upon the extrusion temperature (Table S2).

Besides digestibility, amino acid composition of cooked protein food can differ greatly from that of its raw state due to leaching of soluble protein fractions into the boiling liquid and through the formation of amino acid derivatives (Carbonaro et al., 1997; Friedman et al., 1984; Nierle, 1985; Struthers, 1981). With an altered amino acid composition, the DIAAS value of the cooked protein, and possibly its limiting amino acid, may differ strongly. Animal‐based proteins are also subjected to protein quality variation as a result of processing. Whey protein is generally considered an excellent protein with a DIAAS superior to 100, but surprisingly, multiple references led to DIAAS values ranging from 78 to 88 for whey‐based products, implying that purity and the recovery process can be significant. This further illustrates the importance of considering processing and cooking conditions when determining DIAAS. The present study gathers a large DIAAS dataset of which only a few DIAAS are obtained from cooked products. In order to build a coherent DIAAS value database, the protein intervention material, preferably cooked, and its processing conditions should be well characterized to ensure transparency on the nutritional quality provided to human consumption.

Although findings based on hulls, brans, and peels were excluded, remarkable difference can be observed in the purity of the protein materials studied. Flours contain more fibers than protein isolates or concentrates, thereby also influencing the protein digestibility (Mosenthin, Sauer, & Ahrens, 1994; Myrie, Bertolo, Sauer, & Ball, 2008). It is suggested that protein and nonstarch polysaccharides increase production of pancreatic juices and bile, and stimulate secretion of gut mucin protein composing the mucosal layer, thus contributing to higher ileal AA endogenous losses (Low, 1989; Morel, Melai, Eady, & Coles, 2005). This indicates that applying endogenous loss obtained from a N‐free diet control group to the intervention group could cause an underestimation of the amino acid SID of the studied protein material. True ileal digestibility (TID), also referred to as real ileal digestibility, accounts for diet‐specific endogenous AA loss and is therefore more accurate. However, data based on TID remain scarce due to methodological complications involving such measurement.

Variations observed within each DIAAS dataset can also originate from the conditions in which the intervention diets were provided. Firstly, clear dissimilarities can be noted in starting age/body weight of the growing pigs, varying from 17.8 ± 1.7 kg (Zhang et al., 2015) to 76.2 ± 5.6 kg (Pedersen, Boersma, & Stein, 2007). Several publications (Cunningham, Friend, & Nicholson, 1962; Susenbeth, Dickel, Diekenhorst, & Höhler, 1999) reported a lower CP digestibility in early growing pigs compared with both growing and finishing‐growing pigs. Likewise, younger gestating sows showed a lower apparent crude protein digestibility than older gestating sows (Jacyno et al., 2016). Based on similar results, Hennig and colleagues (Hennig, Bock, Wünsche, & Kreienbring, 1979) suggested that endogenous loss could be of influence. This hypothesis was confirmed by Nitrayova et al. (Nitrayová, Brestenský, Patráš, & Heger, 2013), who reported an increased AA TID concomitantly with a lower AID in pigs of 20.6 kg compared to pigs with average weight of 64.7 kg. Young animals thus tend to produce higher endogenous AA loss than older pigs. Since SID is obtained by correcting AID with endogenous AA losses, SID coefficients will consequently increase in younger animals. This suggests that neonatal pig and piglets could be more suitable models to calculate the DIAAS values of the infant group than growing pigs (Buddington, Ja, Puchal‐Gardiner, & Sangild, 2001; Moughan, Birtles, Cranwell, Smith, & Pedraza, 1992).

Secondly, the daily amount of protein feed provided to the animals varies among studies. For instance, Cervantes‐Pahm (Cervantes‐Pahm et al., 2014) provided two times the maintenance energy requirement during intervention, while the diets in Liu et al. (Liu, Jaworski, Rojas, & Stein, 2016) and Berrocoso et al. (Berrocoso et al., 2015) included 3.4 times the estimated requirement. Rayadurg and Stein (Rayadurg & Stein, 2003) reported a linear decrease in ileal endogenous losses of CP and most AAs as the diet increased from 1 to 3 times the maintenance requirement of growing barrows. Such reduced endogenous ileal AA losses will lead to a greater SID of AAs (Moter & Stein, 2004), eventually impacting the DIAAS value. This further highlights the importance of providing similar animal management conditions to obtain objective DIAAS values. Moreover, three major animal feed databases (CVB, 2016; NRC, 2012; Sauvant, Perez, & Tran, 2004) were included in our datasets, but distinctions between growing pigs, sows, finishing pigs, or piglets are not always detailed, and this also likely contributes to the variation observed in the collected data.

Overall, the differences in the setup of intervention studies highlight the need for harmonized methods when determining growing pig SID. Harmonization might reduce the variation observed in the current investigation and would generate more reliable data for plant proteins. These adjustments should be accompanied by a more detailed characterization of intervention material to ensure reliable DIAAS comparison between protein sources intended to human consumption. Ideally, each food protein source should be classified into subcategories detailing their content in fibers, content of ANFs, and specific processing conditions.

### 4.2 Distinctive DIAAS between protein sources

Independent of the absolute variation in DIAAS observed for each protein source, the overall dataset reveals clear disparities in DIAAS between plant proteins. DIAAS of potato protein reaches the excellent protein quality range (DIAAS ≥100) as defined by FAO, similar to most animal‐derived proteins. Soy and whey proteins obtain a DIAAS score above 75, defining them as high‐quality protein. Corn, wheat, rice, fava bean, oat, and hemp proteins obtain average scores below 60. Plant proteins therefore clearly differ in terms of nutritional profile, and nuances should accordingly be applied when comparing plant proteins to animal‐derived proteins.

Digestible Indispensable Amino Score value is determined by the most limiting digested IAA of the protein. IAA composition of a protein source thus has the majority influence on its DIAAS value. The discrepancies displayed in Figure 2 are for the most part inherent to the IAA content of the protein and are clearly illustrated by gelatin's score. High IAA content combined with low ileal digestibility can considerably impact DIAAS values, particularly if this involves the most commonly limiting IAAs, Lys, or Met + Cys. Ileal digestibility is influenced by several features of the protein. The amino acid sequence may influence the rate of hydrolysis by digestive proteases. Gastric pepsin is known for its affinity with hydrophobic sites (Fruton, 1970) and its propensity to cleave after phenylalanine and leucine residues (Powers, Harley, & Myers, 1977), while pancreatic trypsin favors basic amino acids (arginine and lysine) (Evnin, Vásquez, & Craik, 1990). Moreover, the protein secondary conformations seem to hinder the accessibility of digestive proteases to cleaving sites. High content of beta‐sheet has been reported in soy protein (>40%) (Herrero, Jiménez‐Colmenero, & Carmona, 2009), peas (>40%) (Beck, Knoerzer, & Arcot, 2017), rice (44,9%) (Wang, Wang, Wang, & Chen, 2016), wheat gluten (50%) (Tang et al., 2019), and oat (36,8%–74%) (Liu et al., 2009; Zhao et al., 2017), while such structural arrangement tend to account for less than 30% in milk‐based protein (Carbonaro, Maselli, & Nucara, 2012; Curley, Kumosinski, Unruh, & Farrell, 1998) and meat proteins (Herrero, Carmona, Lopez‐Lopez, & Jimenez‐Colmenero, 2008). This relatively high proportion of beta‐sheet conformations in leguminous and cereal‐based proteins results in reduced protein in vitro digestibility (Carbonaro et al., 2012; Yang et al., 2016). Furthermore, multiple compounds present in plant proteins can interact with digestive enzyme by forming stable complexes and impairing the enzymatic functionality, thereby inhibiting protein digestion (Sarwar Gilani et al., 2012). As detailed earlier, it can be prevented or reduced by suitable processing in the form of heat‐induced denaturation, enzymatic reaction, or fermentation (Sá et al., 2019; Sarwar Gilani et al., 2012).

The vast majority of data used in this investigation are from animal nutrition studies, that is, protein preparations intended for animal feed. Protein products intended for human nutrition may differ in their processing and overall composition. New data on protein material that is specifically intended for food consumption are therefore needed to confidently apply their DIAAS value to human consumption.

### 4.3 Seeking nutritional efficiency

The cause of disparities in DIAAS values appears multifactorial. Depending on the processing conditions, purity of the material, or feeding conditions, a protein DIAAS will vary, making the choice of a representative product per protein source challenging. To further compare the DIAAS of protein sources, we therefore took an objective approach by considering the mean DIAAS values for the 0.5‐ to 3‐year‐old population group. Although DIAAS expresses the quality of protein based on its most limiting IAA score, it also highlights the opportunity to combine the strength of different protein sources. The increased DIAAS values obtained from mixtures show the potential to achieve a protein nutritional efficiency with sustainable protein sources. Nutritional efficiency lies in meeting physiological requirements with minimal intake of high‐quality protein, as opposed to higher protein intake of low‐quality protein. This last scenario is not necessarily favorable due to a potentially high satiety effect resulting in inadequate protein intake and suboptimal sustainability impact of such a diet. Potato protein has shown to be the most promising source of plant protein to achieve nutritional efficiency: With high scores obtained for every IAA, it can boost most mixtures to the excellent protein quality category (DIAAS ≥100). Similarly, soy protein and pea proteins provide good potential to boost many cereal proteins toward the high‐quality protein category (DIAAS = 75–99).

While simulating protein mixtures, crude protein content was not taken into consideration due to the considerable variation among and between proteins. Equations (6) and (8) thus assume an identical protein content between protein sources to ensure the ratio would solely be influenced by IAA content and SID of respective proteins. It is, however, advised to apply the specific CP of proteins in a case‐by‐case manner for the determination of DIAAS of a diet or developing food products. Many of the studies selected for these calculations include protein sources with low average CP content such as oats (18.3%), peas (26%), or fava beans (26.5%), contrary to potato protein (78.9%) and soy protein (49.5%). Mixtures based on potato protein and soy protein therefore tend to generate mixtures richer in protein, contributing to better nutritional efficiency.

The DIAAS method delivers a score characterizing the quality of protein sources. To date, most scores are obtained from single protein materials, while the protein digestibility is known to be influenced by food matrices and food preparation (Dupont, Le Feunteun, Marze, & Souchon, 2018). Lysine, one of the most commonly limiting IAAs, is also one of the most reactive AAs (Hurrell, Carpenter, Sinclair, Otterburn, & Asquith, 1976). During thermal processing, chemical reactions between reducing sugars and lysine lead to formation of Maillard reaction products. Such reactivity reduces the availability of lysine (Nyakayiru et al., 2020), hence the FAO recommendation for the use of digestible reactive lysine instead of total digestible lysine to determine the DIAAS. Such considerations will greatly affect the DIAAS value of protein, and possibly its limiting AA. For example, Hulshof et al. (Hulshof, Bikker, van der Poel, & Hendriks, 2016) observed a 30%–40% decrease in reactive lysine in soybean meal and rapeseed meal after a toasting step in the presence of monosaccharides. A limitation of our study is the reliance on total digestible lysine, as this is most commonly reported in the literature. It is therefore also advised to investigate DIAAS values of processed and/or cooked foods and consider their respective digested reactive lysine to obtain a complete and reliable DIAAS database. Furthermore, in recent years attention has increasingly focused on digestion kinetics and muscle anabolic properties of proteins, triggered by bioavailable leucine (Casperson, Sheffield‐Moore, Hewlings, & Paddon‐Jones, 2012), but DIAAS values do not provide information on these features. However, a higher DIAAS score does suggest good potential for increased net body protein utilization, although the latter must be confirmed by intervention studies tracking net postprandial utilization of prepared foods combining various protein sources. Lastly, dietary protein quality should be further evaluated along with their sustainability impact. The relative contribution of dietary protein in meeting physiological amino acid requirement should perhaps be expressed according to their environmental footprint, as recently highlighted by Tessari et al. (Tessari, Lante, & Mosca, 2016).

## 5 CONCLUSIONS

In conclusion, this investigation shows that protein quality based on DIAAS differs not only between animal and plant proteins but also between plant protein sources. Based on the 0.5‐ to 3‐year‐old scoring pattern, potato and most animal‐derived proteins tend to reach the excellent protein quality category (DIAAS ≥100). Soy and whey proteins fall into the high‐quality protein range (DIAAS = 75–99). Gelatin, corn, wheat, hemp, fava bean, oat, pea, canola, rapeseed, lupin, and rice proteins lie in the no quality claim category (DIAAS <75). Such scores differ greatly for the infant population, for which only casein is of good quality in the current selection. The differences observed in the protein quality lead to opportunities to enhance their nutritional efficiency in the form of protein mixtures. Any protein material can reach a higher DIAAS value when combined with an adequate complementary source. In the current selection, potato, soy, and pea proteins seem the most promising plant‐based complementary sources to reach high or excellent quality mixtures to support a plant‐based lifestyle. Clear dissimilarities were observed within each protein dataset suggesting that: (a) harmonized methods to evaluate protein ileal digestibility; (b) proper characterization of (cooked) protein material; and (b) digestible reactive lysine data are needed to obtain a coherent DIAAS database.

## CONFLICTS OF INTEREST

L.H., P.N., B.P., and M.C.L. are employees of Avebe U.A.

## Supporting information

Tab S1‐S4
