# Supplementary material for: Comprehensive overview of the quality of plant‐ And animal‐sourced proteins based on the digestible indispensable amino acid score
Source: Food Sci Nutr. 2020 Aug 25;8(10):5379–91. doi: 10.1002/fsn3.1809 (PMC7590266; doi:10.1002/fsn3.1809)
Supplement: Supplementary file 1 — Tab S1‐S4 [file FSN3-8-5379-s001.docx]

Table S1. Overview of DIAAS obtained for each dataset. DIAAS calculated for each reference pattern scores as defined by FAO (2013): infant (0-0.5 year old), children (0.5-3 years old) and children older than 3 years old, adolescents and adults.

| **Protein source** | **Protein %** | **Reference** | **DIAAS** | | |
| --- | --- | --- | --- | --- | --- |
|  |  |  | **0-0,5 y** | **0,5-3 y** | **> 3y** |
| Wheat gluten feed | 14.7 | CVB, 2016 | 36 | 44 | 52 |
| Wheat gluten feed | 14.7 | Sauvant et al,. 2004 | 28 | 34 | 40 |
| Wheat flour | 11.67 | Mathai et al., 2017 | 37 | 45 | 54 |
| Wheat hard red | 14.46 | NRC, 2012 | 32 | 39 | 46 |
| Wheat soft red | 10.92 | NRC, 2012 | 38 | 46 | 55 |
| Wheat gluten | 72.11 | NRC, 2012 | 20 | 25 | 29 |
| Wheat | 11.19 | Wang et al., 2018 | 33 | 40 | 48 |
| Wheat | 11.9 | Cervantes-Pahm et al., 2014 | 30 | 37 | 43 |
| Wheat | 10.5 | McGhee and Stein, 2018 | 37 | 45 | 54 |
| Wheat | 11.3 | Lee et al., 2019 | 39 | 49 | 59 |
| Wheat soft | 10.5 | Sauvant et al., 2004 | 34 | 41 | 49 |
| Wheat feed flour | 12.7 | Sauvant et al., 2004 | 48 | 58 | 69 |
| Wheat gluten feed - 28% starch | 14.5 | Sauvant et al., 2004 | 28 | 34 | 40 |
| Wheat gluten feed - 25% starch | 14.7 | Sauvant et al., 2004 | 41 | 50 | 59 |
| Wheat | 11 | CVB, 2016 | 34 | 41 | 49 |
| Wheat feed flour CF< 35 g/kg | 15.4 | CVB, 2016 | 52 | 62 | 74 |
| Wheat feed flour CF>35 g/kg | 15.5 | CVB, 2016 | 49 | 59 | 70 |
| Wheat feed meal | 15.3 | CVB, 2016 | 45 | 54 | 64 |
| Wheat germ feed | 17.9 | CVB, 2016 | 47 | 57 | 68 |
| Wheat germs | 26.4 | CVB, 2016 | 52 | 78 | 85 |
| Wheat gluten feed <40g/k | 14.7 | CVB, 2016 | 36 | 44 | 52 |
| Wheat gluten feed <50-60g/kg | 16 | CVB, 2016 | 36 | 44 | 52 |
| Wheat gluten feed >60g/kg | 15.5 | CVB, 2016 | 36 | 44 | 52 |
| Wheat gluten meal | 78.1 | CVB, 2016 | 24 | 30 | 35 |
| Triticale | 10.7 | CVB,2016 | 40 | 48 | 57 |
| Wheat Cultivar 1 | 14.22 | Zhao et al., 2018 | 40 | 48 | 57 |
| Wheat Cultivar 2 | 13.65 | Zhao et al., 2018 | 43 | 52 | 61 |
| Wheat Cultivar 5 | 14.65 | Zhao et al., 2018 | 44 | 53 | 63 |
| Wheat Cultivar 6 | 14.5 | Zhao et al., 2018 | 45 | 54 | 64 |
| Wheat Cultivar 7 | 17.46 | Zhao et al., 2018 | 36 | 44 | 52 |
| Wheat Cultivar 11 | 18.14 | Zhao et al., 2018 | 40 | 49 | 58 |
| Wheat Cultivar 13 | 14.41 | Zhao et al., 2018 | 43 | 52 | 62 |
| Wheat Cultivar 14 | 14.43 | Zhao et al., 2018 | 38 | 49 | 58 |
| Wheat Cultivar 15 | 14.62 | Zhao et al., 2018 | 47 | 64 | 76 |
| Wheat Cultivar 16 | 16.02 | Zhao et al., 2018 | 41 | 50 | 59 |
| Wheat | 13.5 | Woyengo et al., 2014 | 36 | 44 | 52 |
| Wheat | 12.44 | Pedersen et al., 2007 | 31 | 38 | 45 |
| Rice feed meal – ash < 90 kg/g | 13.7 | CVB, 2016 | 38 | 46 | 54 |
| Rice feed meal – ash > 90 kg/g | 13.9 | CVB, 2016 | 38 | 46 | 54 |
| Rice protein concentrate | 67.5 | Gottlob et al., 2006 | 41 | 50 | 59 |
| Oats peeled | 13.2 | CVB, 2016 | 51 | 62 | 73 |

Table S1.*(continued)*

| **Protein source** | **Protein %** | **Reference** | **DIAAS** | | |
| --- | --- | --- | --- | --- | --- |
|  |  |  | **0-0,5 y** | **0,5-3 y** | **> 3y** |
| Oat groats | 10.6 | Sauvant et al., 2004 | 48 | 58 | 69 |
| Oat groats | 13.9 | NRC, 2012 | 40 | 48 | 57 |
| Oats | 10.2 | CVB, 2016 | 48 | 58 | 68 |
| Oat protein concentrate | 59.7 | Abelilla et al., 2017 | 41 | 50 | 59 |
| Dehulled oats | 13.1 | Cervantes-Pahm et al., 2014 | 37 | 65 | 77 |
| Oats | 11.16 | NRC, 2012 | 48 | 59 | 70 |
| Oats naked | 14.7 | NRC, 2012 | 50 | 60 | 71 |
| Rapeseed full fat | 19.1 | Sauvant et al., 2004 | 47 | 69 | 74 |
| Rapeseed | 19.8 | CVB, 2016 | 52 | 70 | 84 |
| Rapeseed solvant extracted | 38.8 | CVB, 2016 | 53 | 71 | 85 |
| Rapeseed meal | 33.7 | Sauvant et al., 2004 | 56 | 70 | 83 |
| Rapeseed meal 1 | 36.35 | Maison et al., 2014 | 47 | 63 | 75 |
| Rapeseed meal 2 | 38.02 | Maison et al., 2014 | 51 | 67 | 79 |
| Rapeseed meal 3 | 37.52 | Maison et al., 2014 | 55 | 76 | 89 |
| Rapeseed meal 4 | 35.64 | Maison et al., 2014 | 51 | 71 | 84 |
| Rapeseed meal 5 | 35.85 | Maison et al., 2014 | 42 | 59 | 70 |
| Rapeseed meal 6 | 36.52 | Maison et al., 2014 | 51 | 66 | 79 |
| Rapeseed meal 7 | 37.11 | Maison et al., 2014 | 50 | 75 | 87 |
| Rapeseed meal 8 | 37.3 | Maison et al., 2014 | 49 | 60 | 71 |
| Rapeseed meal 9 | 35.63 | Maison et al., 2014 | 46 | 65 | 77 |
| Rapeseed meal 10 | 37.07 | Maison et al., 2014 | 52 | 65 | 77 |
| Rapeseed expellers 1 | 36.13 | Maison et al., 2014 | 47 | 88 | 88 |
| Rapeseed expellers 2 | 34.51 | Maison et al., 2014 | 51 | 81 | 88 |
| Rapeseed expellers 3 | 36.22 | Maison et al., 2014 | 55 | 65 | 78 |
| Rapeseed expellers 4 | 35.21 | Maison et al., 2014 | 51 | 68 | 81 |
| Rapeseed expellers 5 | 35.82 | Maison et al., 2014 | 42 | 56 | 67 |
| Double low rapeseed meal 1 | 42.67 | Li et al., 2015 | 43 | 54 | 64 |
| Double low rapeseed meal 2 | 43.59 | Li et al., 2015 | 44 | 54 | 64 |
| Double low rapeseed meal 3 | 41.35 | Li et al., 2015 | 42 | 60 | 71 |
| Double low rapeseed meal 4 | 43.64 | Li et al., 2015 | 43 | 73 | 83 |
| Double low rapeseed meal 5 | 42.26 | Li et al., 2015 | 44 | 68 | 80 |
| Double low rapeseed meal 8 | 43.29 | Li et al., 2015 | 41 | 53 | 63 |
| Double low rapeseed meal 10 | 43.02 | Li et al., 2015 | 43 | 60 | 72 |
| Double low rapeseed meal 11 | 40.29 | Li et al., 2015 | 43 | 57 | 68 |
| Double low rapeseed meal 12 | 41.48 | Li et al., 2015 | 43 | 72 | 80 |
| Double low rapeseed meal | 38.3 | Hulshof et al., 2016 | 56 | 75 | 89 |
| Processed Double low rapeseed meal | 37.1 | Hulshof et al., 2016 | 36 | 43 | 52 |
| Rapeseed meal | 38.75 | Huang et al., 2018 | 43 | 76 | 90 |
| Peas | 20.3 | CVB, 2016 | 37 | 66 | 77 |
| Pea seed | 20.7 | Sauvant et al., 2004 | 39 | 64 | 78 |
| Pea protein concentrate | 54.46 | Mathai et al., 2017 | 45 | 63 | 74 |
| Field peas | 22.17 | NRC, 2012 | 38 | 63 | 74 |

Table S1.*(continued)*

| **Protein source** | **Protein %** | **Reference** | **DIAAS** | | |
| --- | --- | --- | --- | --- | --- |
|  |  |  | **0-0,5 y** | **0,5-3 y** | **> 3y** |
| Pea - Baccara 1 | 25.48 | Grosjean et al., 2000 | 48 | 59 | 69 |
| Pea - Baccara 2 | 26.11 | Grosjean et al., 2000 | 46 | 56 | 66 |
| Pea - Victor 1 | 23.18 | Grosjean et al., 2000 | 57 | 69 | 81 |
| Pea - Brévent | 26.71 | Grosjean et al., 2000 | 58 | 71 | 83 |
| Pea - Victor 2 | 25.52 | Grosjean et al., 2000 | 55 | 67 | 79 |
| Pea - Blizard | 246 | Grosjean et al., 2000 | 44 | 54 | 63 |
| Pea - Névé | 24.4 | Grosjean et al., 2000 | 53 | 64 | 76 |
| Pea - Cheyenne | 21.64 | Grosjean et al., 2000 | 63 | 77 | 91 |
| Pea - Froidure | 24.27 | Grosjean et al., 2000 | 52 | 63 | 74 |
| Pea - Aravis | 24.76 | Grosjean et al., 2000 | 53 | 65 | 77 |
| Pea - Rafale | 27.73 | Grosjean et al., 2000 | 50 | 61 | 72 |
| Pea - Radley | 24.56 | Grosjean et al., 2000 | 56 | 69 | 81 |
| Pea - Baroness | 23.79 | Grosjean et al., 2000 | 33 | 65 | 84 |
| Pea | 17.72 | Stein and Bohlke, 2007 | 35 | 70 | 90 |
| Extruded Pea - 75°C | 18.05 | Stein and Bohlke, 2007 | 43 | 86 | 109 |
| Extruded Pea - 115°C | 19.17 | Stein and Bohlke, 2007 | 42 | 85 | 100 |
| Extruded Pea - 155°C | 18.5 | Stein and Bohlke, 2007 | 41 | 82 | 106 |
| Pelleted Pea - 75°C | 17.76 | Stein and Bohlke, 2007 | 33 | 65 | 84 |
| Processed Soybean meal | 51.1 | Hulshof et al., 2016 | 45 | 54 | 64 |
| Soybean meal | 53.1 | Hulshof et al., 2016 | 64 | 87 | 102 |
| Soybean meal dehulled Korea 1 | 47.1 | Son et al., 2019 | 48 | 59 | 69 |
| Soybean meal dehulled Korea 2 | 47.4 | Son et al., 2019 | 54 | 81 | 87 |
| Soybean meal India | 39.6 | Son et al., 2019 | 54 | 66 | 78 |
| Soy bean meal | 45.6 | Kong et al., 2014 | 36 | 71 | 92 |
| Soybean full fat extruded | 34.80 | Sauvant et al., 2004 | 59 | 94 | 101 |
| Soybean full fat toasted | 35.2 | Sauvant et al., 2004 | 59 | 83 | 89 |
| Soybean meal 46 | 43.3 | Sauvant et al., 2004 | 68 | 96 | 106 |
| Soybean meal 48 | 45.3 | Sauvant et al., 2004 | 68 | 96 | 106 |
| Soybeans, Low Oligosaccharide, Full Fat | 39.3 | NRC, 2012 | 73 | 101 | 112 |
| Soya beans, heat treated | 36.3 | CVB,2016 | 63 | 84 | 94 |
| Soybean meal solvent extracted-HiPro CF < 45 g/kg-CP > 490 g/kg | 48.5 | CVB. 2016 | 68 | 96 | 106 |
| Soybean expeller | 43.8 | CVB, 2016 | 67 | 93 | 104 |
| Soybean meal 50 | 47.2 | Sauvant et al., 2004 | 69 | 98 | 108 |
| Soy protein isolate | 92.66 | Mathai et al., 2017 | 68 | 84 | 98 |
| Soy flour | 52.29 | Mathai et al., 2017 | 72 | 89 | 105 |
| Soybean meal | 47.11 | Liu et al., 2014 | 29 | 84 | 51 |
| Soybean meal | 49.5 | Liu et al., 2016 | 68 | 83 | 97 |
| Soybean meal | 48.3 | Berrocoso et al., 2015 | 73 | 95 | 111 |
| High protein full fat soy beans (FFSB-HP) | 47.6 | Cervantes-Pahm et al., 2008 | 37 | 73 | 95 |

Table S1.*(continued)*

| **Protein source** | **Protein %** | **Reference** | **DIAAS** | | |
| --- | --- | --- | --- | --- | --- |
|  |  |  | **0-0,5 y** | **0,5-3 y** | **> 3y** |
| Conventional full fat soybeans (FFSB-CV) | 35.8 | Cervantes-Pahm et al., 2008 | 49 | 97 | 125 |
| Soybean meal (SBM) | 42.9 | Cervantes-Pahm et al., 2008 | 66 | 93 | 100 |
| Soy protein concentrate (SPC) | 64.2 | Cervantes-Pahm et al., 2008 | 69 | 97 | 112 |
| Extracted SBM-HP | 55.65 | Baker and Stein, 2009 | 71 | 96 | 107 |
| Extracted SBM-CONV | 48.36 | Baker and Stein, 2009 | 72 | 97 | 110 |
| Extracted expelled SBM-HP | 55.97 | Baker and Stein, 2009 | 68 | 88 | 104 |
| Extracted expelled SBM-LO | 49.33 | Baker and Stein, 2009 | 73 | 97 | 111 |
| Extracted expelled SBM-CONV | 47.09 | Baker and Stein, 2009 | 73 | 89 | 105 |
| Soybean Meal, Dehulled, Expelled | 45.13 | NRC, 2012 | 65 | 91 | 98 |
| Soybean Meal, Dehulled, Solvent Extracted | 47.73 | NRC, 2012 | 70 | 92 | 102 |
| Soybean Meal, Enzyme Treated | 55.62 | NRC, 2012 | 63 | 81 | 96 |
| Soybean Meal, Expelled | 44.56 | NRC, 2012 | 68 | 91 | 102 |
| Soybean Meal, Fermented | 54.07 | NRC, 2012 | 59 | 76 | 91 |
| Soybean Meal, High Protein, Dehulled, Solvent Extracted | 51.17 | NRC, 2012 | 66 | 89 | 102 |
| Soybean Meal, High Protein, Expelled | 55.97 | NRC, 2012 | 69 | 89 | 104 |
| Soybean Meal, Low Oligosaccharide, Expelled | 49.33 | NRC, 2012 | 73 | 97 | 112 |
| Soybean Meal, Solvent Extracted | 43.9 | NRC, 2012 | 67 | 86 | 92 |
| Soybeans, Full Fat | 37.56 | NRC, 2012 | 58 | 82 | 89 |
| Soybeans, High Protein, Full Fat | 42.77 | NRC, 2012 | 59 | 86 | 93 |
| Soy Protein Concentrate | 65.2 | NRC, 2012 | 64 | 86 | 101 |
| Soy Protein Isolate | 84.78 | NRC, 2012 | 62 | 75 | 88 |
| Soy bean meal | 44.8 | Lee et al., 2018 | 56 | 99 | 106 |
| Whey powder | 13.0 | CVB, 2016 | 55 | 81 | 101 |
| Whey powder, low lactose | 21.7 | CVB. 2016 | 56 | 83 | 104 |
| Cheese whey | 15.0 | CVB. 2016 | 50 | 72 | 90 |
| Cheese whey | 20.9 | CVB, 2016 | 50 | 72 | 90 |
| Cheese whey | 33.5 | CVB, 2016 | 50 | 72 | 90 |
| Whey powder, low lactose | 25.2 | CVB, 2016 | 56 | 83 | 104 |
| Whey powder sweet | 12.6 | Sauvant et al., 2004 | 43 | 78 | 99 |
| Whey protein isolate | 85.2 | Mathai et al., 2017 | 67 | 100 | 125 |
| Whey protein concentrate | 78.01 | Mathai et al., 2017 | 71 | 107 | 134 |
| Whey protein concentrate | 80.18 | Gottlob et al., 2006 | 59 | 88 | 111 |
| Whey protein concentrate | 76.32 | NRC, 2012 | 61 | 88 | 110 |
| Whey powder | 11.55 | NRC, 2012 | 53 | 87 | 109 |
| Casein | 87.2 | CVB, 2016 | 75 | 120 | 141 |
| Casein | 88.95 | NRC 2012 | 84 | 113 | 132 |
| Spray-dried egg | 42.76 | Zhang et al., 2015 | 51 | 85 | 91 |
| Albumen powder | 73.23 | Zhang et al., 2015 | 65 | 79 | 93 |

Table S1.*(continued)*

| **Protein source** | **Protein %** | **Reference** | **DIAAS** | | |
| --- | --- | --- | --- | --- | --- |
|  |  |  | **0-0,5 y** | **0,5-3 y** | **> 3y** |
| Pasteurized whole egg | 47.1 | Woyengo et al., 2015 | 83 | 110 | 133 |
| Canola | 40.2 | Wang et al., 2018 | 56 | 69 | 82 |
| High protein canola meal 1 | 44.9 | Berrocoso et al., 2015 | 58 | 82 | 98 |
| High protein canola meal 2 | 47.5 | Berrocoso et al., 2015 | 57 | 80 | 95 |
| Conventional canola meal | 36.8 | Berrocoso et al., 2015 | 54 | 75 | 89 |
| High-protein canola meal | 44.72 | Liu et al., 2014 | 22 | 76 | 39 |
| High-temperature processed canola meal | 36.02 | Liu et al., 2014 | 21 | 74 | 36 |
| Low-temperature processed canola meal | 36.99 | Liu et al., 2014 | 21 | 74 | 37 |
| Conventional canola meal | 34.2 | Liu et al., 2014 | 18 | 65 | 32 |
| High protein canola meal | 45.0 | Liu et al., 2016 | 51 | 76 | 83 |
| Conventional canola meal | 40.2 | Liu et al., 2016 | 53 | 78 | 84 |
| Full-fat canola seeds | 24.8 | Park et al., 2019 | 54 | 58 | 86 |
| Canola meal | 43.6 | Park et al., 2019 | 52 | 57 | 85 |
| Canola expellers | 36.5 | Park et al., 2019 | 56 | 59 | 91 |
| Canola, Full Fat | 22.06 | NRC 2012 | 37 | 59 | 64 |
| Canola Meal, Expelled | 35.19 | NRC 2012 | 39 | 56 | 66 |
| Canola Meal, Solvent Extracted | 37.5 | NRC 2012 | 48 | 72 | 84 |
| Canola Meal 1 | 39.35 | Maison et al., 2014 | 54 | 73 | 87 |
| Canola Meal 2 | 36.74 | Maison et al., 2014 | 51 | 72 | 85 |
| Canola Meal 3 | 39.77 | Maison et al., 2014 | 51 | 64 | 76 |
| Canola Meal 4 | 38.08 | Maison et al., 2014 | 51 | 65 | 77 |
| Canola Meal 5 | 36.7 | Maison et al., 2014 | 51 | 62 | 73 |
| Canola Meal 6 | 37.56 | Maison et al., 2014 | 46 | 63 | 75 |
| Canola Meal 7 | 36.59 | Maison et al., 2014 | 48 | 58 | 69 |
| Canola meal | 37.5 | Son et al., 2019 | 42 | 51 | 60 |
| Canola meal | 42.85 | Xue et al., 2014 | 58 | 83 | 98 |
| Canola meal | 38.5 | Seneviratne et al., 2010 | 49 | 81 | 87 |
| Maize gluten feed | 24.0 | CVB, 2016 | 23 | 34 | 41 |
| Maize gluten feed | 20.3 | CVB, 2016 | 23 | 34 | 41 |
| Maize germ meal feed expeller | 13.4 | CVB, 2016 | 24 | 35 | 41 |
| Maize germ meal feed, solvent extracted | 13.8 | CVB, 2016 | 24 | 35 | 41 |
| Maize germ meal, solvent extracted | 19.9 | CVB, 2016 | 26 | 33 | 39 |
| Maize gluten meal | 59.9 | CVB, 2016 | 21 | 26 | 31 |
| Corn gluten meal | 60.6 | Sauvant et al,. 2004 | 23 | 28 | 33 |
| Corn gluten feed | 17.39 | NRC, 2012 | 16 | 31 | 40 |
| Corn gluten meal | 58.25 | NRC, 2012 | 21 | 34 | 40 |
| Corn gluten meal | 62.9 | Almeida et al., 2011 | 22 | 27 | 32 |
| Corn gluten feed China | 21.56 | Lee et al., 2019 | 13 | 27 | 34 |
| Corn gluten feed Korea | 24.55 | Lee et al., 2019 | 6 | 25 | 30 |

Table S1.*(continued)*

| **Protein source** | **Protein %** | **Reference** | **DIAAS** | | |
| --- | --- | --- | --- | --- | --- |
|  |  |  | **0-0,5 y** | **0,5-3 y** | **> 3y** |
| Corn gluten meal | 65.25 | Lee et al., 2019 | 19 | 25 | 30 |
| Corn gluten meal 1 | 49.8 | Ji et al., 2012 | 17 | 21 | 25 |
| Corn gluten meal 2 | 57.2 | Ji et al., 2012 | 17 | 21 | 25 |
| Corn gluten meal 3 | 59.2 | Ji et al., 2012 | 17 | 21 | 25 |
| Corn gluten meal 4 | 59.7 | Ji et al., 2012 | 18 | 21 | 26 |
| Corn gluten meal 5 | 54.5 | Ji et al., 2012 | 18 | 22 | 26 |
| Corn gluten meal 6 | 64.1 | Ji et al., 2012 | 18 | 21 | 25 |
| Corn gluten meal 7 | 69.6 | Ji et al., 2012 | 16 | 20 | 24 |
| Corn gluten meal 8 | 63.7 | Ji et al., 2012 | 20 | 25 | 29 |
| Corn gluten meal 9 | 65.8 | Ji et al., 2012 | 22 | 27 | 32 |
| Corn gluten meal 10 | 71.2 | Ji et al., 2012 | 20 | 25 | 29 |
| Corn gluten meal 11 | 51 | Ji et al., 2012 | 17 | 21 | 24 |
| Corn gluten meal 12 | 50.5 | Ji et al., 2012 | 19 | 23 | 27 |
| Corn gluten meal 13 | 67.8 | Ji et al., 2012 | 19 | 23 | 27 |
| Corn gluten meal 14 | 70.7 | Ji et al., 2012 | 19 | 22 | 27 |
| Corn gluten meal 15 | 64.2 | Ji et al., 2012 | 17 | 21 | 25 |
| Maize germ meal solvent extracted | 25.8 | Sauvant et al., 2004 | 24 | 29 | 34 |
| Corn gluten feed | 19.3 | Sauvant et al., 2004 | 23 | 35 | 41 |
| Corn germ | 14.79 | NRC 2012 | 25 | 50 | 59 |
| Corn germ meal | 23.33 | NRC 2012 | 26 | 38 | 41 |
| Corn | 10.95 | Xue et al., 2014 | 35 | 53 | 63 |
| Corn germ meal 1 | 18.27 | Zhang et al., 2019 | 28 | 52 | 61 |
| Corn germ meal 2 | 18.77 | Zhang et al., 2019 | 36 | 63 | 75 |
| Corn germ meal 3 | 19.64 | Zhang et al., 2019 | 34 | 51 | 61 |
| Corn germ meal 4 | 19.26 | Zhang et al., 2019 | 25 | 46 | 55 |
| Corn germ meal 5 | 22.73 | Zhang et al., 2019 | 22 | 43 | 56 |
| Corn germ meal 6 | 18.09 | Zhang et al., 2019 | 29 | 52 | 62 |
| Corn germ meal 7 | 17.23 | Zhang et al., 2019 | 38 | 63 | 74 |
| Corn germ meal 8 | 19.35 | Zhang et al., 2019 | 32 | 57 | 67 |
| Corn germ meal 9 | 16.97 | Zhang et al., 2019 | 21 | 39 | 47 |
| Corn germ meal 10 | 18.71 | Zhang et al., 2019 | 41 | 57 | 68 |
| Corn germ meal | 21.4 | Son et al., 2019 | 34 | 42 | 50 |
| Potato protein concentrate | 77.6 | Sauvant et al., 2004 | 53 | 98 | 122 |
| Potato protein | 78.5 | CVB, 2016 | 66 | 96 | 120 |
| Potato proteins | 79.5 | CVB, 2016 | 66 | 96 | 120 |
| Potato protein | 81.4 | Beelen et al., 1999 | 72 | 113 | 134 |
| Potato protein concentrate | 79.8 | NRC, 2012 | 64 | 97 | 121 |
| Cold-pressed hemp seed cake | 81.4 | Presto et al., 2011 | 45 | 54 | 64 |
| Pork gelatin | 93.4 | Petersen et al., 2005 | 0 | 0 | 0 |
| Beef gelatin | 94 | Petersen et al., 2005 | 0 | 0 | 0 |
| Gelatin | 100.1 | NRC, 2012 | 5 | 10 | 13 |

Table S1.*(continued)*

| **Protein source** | **Protein %** | **Reference** | **DIAAS** | | |
| --- | --- | --- | --- | --- | --- |
|  |  |  | **0-0,5 y** | **0,5-3 y** | **> 3y** |
| Lupins | 32.44 | NRC, 2012 | 39 | 63 | 74 |
| Lupin kernels | 31.13 | Lee et al., 2019 | 43 | 82 | 97 |
| Lupins CP<335 G/KG | 31.4 | CVB, 2016 | 41 | 69 | 81 |
| Lupins CP>335 G/KG | 36.2 | CVB, 2016 | 41 | 69 | 81 |
| Faba bean | 27.16 | NRC, 2012 | 30 | 49 | 57 |
| Faba bean Coloured flowers | 25.4 | Sauvant et al., 2004 | 33 | 56 | 66 |
| Faba bean White flowers | 26.8 | Sauvant et al., 2004 | 38 | 59 | 69 |
| Pork- Raw belly | 16.46 | Bailey al., 2019 | 57 | 110 | 118 |
| Pork- Smoked-cooked bacon | 19.15 | Bailey al., 2019 | 72 | 109 | 117 |
| Pork- Smoked bacon | 34.13 | Bailey al., 2019 | 63 | 125 | 142 |
| Pork- Non-cured ham | 34.52 | Bailey al., 2019 | 72 | 115 | 123 |
| Pork- Alt. Cured ham | 24.17 | Bailey al., 2019 | 78 | 123 | 132 |
| Pork- Conv cured ham | 23.79 | Bailey al., 2019 | 73 | 116 | 125 |
| Pork- Loin 63°C | 25.58 | Bailey al., 2019 | 87 | 129 | 139 |
| Pork- Loin 68°C | 30.86 | Bailey al., 2019 | 73 | 109 | 118 |
| Pork- Loin 72°C | 33.3 | Bailey al., 2019 | 74 | 109 | 118 |

Table S2. Digestible indispensable amino acid scores of protein sources according to the 0-0.5 years old reference pattern score. Data expressed as mean of individual DIAA values ± standard deviation.

| **Protein source** | **Histidine** | **Isoleucine** | **Leucine** | **Lysine** | **Met + Cys** | **Phe + Tyr** | **Threonine** | **Tryptophan** | **Valine** | **DIAAS** | **Limiting AA** |
| --- | --- | --- | --- | --- | --- | --- | --- | --- | --- | --- | --- |
| Corn | 104 ± 28.3 | 52 ± 8.5 | 112 ± 40 | 30 ± 12.3 | 103 ± 18.1 | 78 ± 23.7 | 60 ± 7.2 | 26 ± 17.7 | 70 ± 11.3 | 26 | Trp |
| Rice | 89 ± 6.7 | 52 ± 10.1 | 55 ± 8.5 | 39 ± 1.9 | 85 ± 9.0 | 66 ± 16.4 | 53 ± 2.9 | 57 ± 14.3 | 74 ± 14.1 | 39 | Lys |
| Wheat | 113 ± 21.2 | 53 ± 6.2 | 60 ± 7.7 | 39 ± 8.6 | 103 ± 18.6 | 60 ± 11.4 | 56 ± 6.0 | 63 ± 9.1 | 72 ± 7.9 | 39 | Lys |
| Hemp | 118 ± NA | 62 ± NA | 58 ± NA | 45 ± NA | 99 ± NA | 72 ± NA | 61 ± NA | - | 77 ± NA | 45 | Lys |
| Fava bean | 103 ± 3.9 | 62 ± 1.3 | 65 ± 3.7 | 79 ± 3.6 | 45 ± 4.2 | 66 ± 1.9 | 64 ± 4.4 | 34 ± 3.9 | 65 ± 1.7 | 34 | Trp |
| Oat | 87 ± 10.9 | 58 ± 2.4 | 65 ± 3.4 | 47 ± 4.8 | 124 ± 43.3 | 74 ± 5.1 | 60 ± 4.2 | 55 ± 8.6 | 80 ± 2.6 | 47 | Lys |
| Rapeseed | 102 ± 8.5 | 53 ± 9 | 55 ± 6.9 | 56 ± 10.2 | 103 ± 13.8 | 53 ± 14.8 | 69 ± 9.3 | 55 ± 15.4 | 72 ± 6.5 | 53 | P+T |
| Lupin | 116 ± 15 | 68 ± 5.7 | 66 ± 6.3 | 64 ± 6.9 | 58 ± 6.8 | 74 ± 5.5 | 73 ± 9.6 | 41 ± 1.9 | 64 ± 7.1 | 41 | Trp |
| Pea | 94 ± 9.2 | 59 ± 7.6 | 60 ± 7.9 | 91 ± 8.9 | 58 ± 10.0 | 64 ± 9 | 66 ± 5.6 | 39 ± 3.5 | 65 ± 7.7 | 39 | Trp |
| Canola | 100 ± 6.5 | 55 ± 6.7 | 55 ± 6.5 | 60 ± 7.0 | 100 ± 7.9 | 55 ± 8.6 | 69 ± 6.6 | 57 ± 11.1 | 69 ± 6.2 | 55 | P+T |
| Soy | 113 ± 8.9 | 72 ± 4.8 | 70 ± 4.2 | 80 ± 7.4 | 74 ± 9.4 | 81 ± 4.6 | 74 ± 4.2 | 66 ± 10.5 | 75 ± 5.7 | 66 | Trp |
| Potato | 96 ± 8 | 90 ± 6.1 | 100 ± 8.1 | 102 ± 3.9 | 93 ± 4.8 | 115 ± 11.1 | 116 ± 9.7 | 67 ± 3.3 | 108 ± 4.5 | 67 | Trp |
| Gelatin | 32 ± 9.0 | 23 ± 1.8 | 27 ± 1.9 | 53 ± 7.2 | 23 ± 6.9 | 23 ± 3.7 | 36 ± 3.4 | 2 ± 3.0 | 39 ± 3.2 | 2 | Trp |
| Whey | 81 ± 10.3 | 96 ± 13.5 | 95 ± 15.7 | 108 ± 20.8 | 108 ± 17.7 | 56 ± 7.7 | 123 ± 16.1 | 90 ± 23.5 | 91 ± 11.2 | 56 | P+T |
| Egg | 96 ± 11.1 | 75 ± 14.8 | 71 ± 11.1 | 110 ± 48.3 | 102 ± 45.6 | 80 ± 10.5 | 74 ± 9.9 | 64 ± 24.9 | 82 ± 25.3 | 64 | Trp |
| Casein | 149 ± 4.4 | 106 ± 21.7 | 100 ± 0.6 | 100 ± 19.4 | 86 ± 17.5 | 135 ± 29.5 | 108 ± 19.5 | 87 ± 17.4 | 100 ± 24.4 | 86 | M+C |
| Pork | 187 ± 13 | 89 ± 6.5 | 84 ± 6.3 | 130 ± 8.8 | 104 ± 8.8 | 82 ± 5.8 | 102 ± 7.1 | 72 ± 8.6 | 91 ± 7.1 | 72 | Trp |

Table S3. Digestible indispensable amino acid scores of protein sources according to the reference pattern score for children older than 3 years old, adolescents and adults. Data expressed as mean of individual DIAA values ± standard deviation.

| **Protein source** | **Histidine** | **Isoleucine** | **Leucine** | **Lysine** | **Met + Cys** | **Phe + Tyr** | **Threonine** | **Tryptophan** | **Valine** | **DIAAS** | **Limiting AA** |
| --- | --- | --- | --- | --- | --- | --- | --- | --- | --- | --- | --- |
| Corn | 137 ± 37.2 | 95 ± 15.6 | 176 ± 63.0 | 43 ± 17.7 | 148 ± 26.0 | 178 ± 54.3 | 106 ± 12.7 | 66 ± 45.6 | 96 ± 15.5 | 43 | Lys |
| Rice | 116 ± 8.8 | 95 ± 18.5 | 87 ± 13.4 | 56 ± 2.8 | 122 ± 12.9 | 151 ± 37.6 | 93 ± 5.1 | 146 ± 36.9 | 102 ± 19.4 | 56 | Lys |
| Wheat | 148 ± 28.1 | 98 ± 11.0 | 95 ± 11.9 | 56 ± 13.8 | 150 ± 22.4 | 138 ± 25.7 | 97 ± 11.4 | 162 ± 25 | 98 ± 11.2 | 56 | Lys |
| Hemp | 116 ± 8.8 | 95 ± 18.5 | 87 ± 13.4 | 56 ± 2.8 | 122 ± 12.9 | 151 ± 37.6 | 93 ± 5.1 | 146 ± 36.9 | 102 ± 19.4 | 56 | Lys |
| Fava bean | 135 ± 5.2 | 114 ± 2.3 | 103 ± 5.8 | 113 ± 5.1 | 64 ± 6.0 | 150 ± 4.3 | 113 ± 7.7 | 87 ± 10.0 | 90 ± 2.4 | 64 | M+C |
| Oat | 114 ± 14.3 | 106 ± 4.4 | 102 ± 5.3 | 68 ± 6.8 | 177 ± 62.1 | 171 ± 11.7 | 106 ± 7.3 | 142 ± 22.2 | 110 ± 3.6 | 68 | Lys |
| Rapeseed | 133 ± 7.4 | 95 ± 4.9 | 85 ± 5.1 | 79 ± 11.1 | 145 ± 16.1 | 116 ± 13.6 | 119 ± 6.9 | 136 ± 9.8 | 98 ± 4.5 | 79 | Lys |
| Lupin | 153 ± 19.7 | 124 ± 10.5 | 104 ± 9.9 | 92 ± 9.9 | 83 ± 9.7 | 170 ± 12.5 | 129 ± 16.8 | 105 ± 4.8 | 88 ± 9.7 | 83 | M+C |
| Pea | 124 ± 12.1 | 108 ± 14 | 94 ± 12.4 | 130 ± 12.8 | 83 ± 14.4 | 148 ± 20.7 | 117 ± 9.8 | 100 ± 9.1 | 89 ± 10.6 | 83 | M+C |
| Canola | 131 ± 8.7 | 99 ± 10.5 | 85.4 ± 8.5 | 85.1 ± 10.9 | 143 ± 12.2 | 123 ± 7.7 | 119 ± 12.2 | 144 ± 25.1 | 93 ± 10.6 | 85 | Lys |
| Soy | 149 ± 11.7 | 133 ± 8.8 | 110 ± 6.6 | 114 ± 10.7 | 106 ± 13.5 | 186 ± 10.6 | 130 ± 7.5 | 170 ± 27.1 | 103 ± 7.9 | 103 | NA |
| Potato | 125 ± 9.1 | 167 ± 9.8 | 155 ± 12.1 | 145 ± 5.5 | 135 ± 7.1 | 266 ± 23.1 | 204 ± 14.8 | 165 ± 17.6 | 149 ± 5.5 | 125 | NA |
| Gelatin | 43 ± 11.8 | 41 ± 3.3 | 42 ± 3.0 | 76 ± 10.3 | 33 ± 9.9 | 53 ± 8.4 | 63 ± 6.0 | 4 ± 7.7 | 53 ± 4.4 | 4 | Trp |
| Whey | 106 ± 13.5 | 173 ± 24 | 145 ± 25.4 | 151 ± 28.6 | 152 ± 26 | 124 ± 20.3 | 213 ± 29.3 | 220 ± 58.1 | 122 ± 15.4 | 106 | NA |
| Egg | 127 ± 14.6 | 137 ± 27.2 | 111 ± 17.5 | 158 ± 69.4 | 146 ± 65.4 | 183 ± 24.0 | 131 ± 17.4 | 166 ± 64 | 113 ± 34.7 | 111 | NA |
| Casein | 183 ± 11.8 | 163 ± 4.6 | 152 ± 7.2 | 160 ± 5.1 | 137 ± 5.9 | 255 ± 10.2 | 161 ± 5.3 | 205 ± 17.3 | 159 ± 2.9 | 137 | NA |
| Pork | 246 ± 17.1 | 163 ± 11.8 | 132 ± 9.9 | 186 ± 12.7 | 150 ± 12.6 | 188 ± 13.2 | 180 ± 12.5 | 185 ± 22 | 126 ± 9.7 | 126 | NA |

Table S4. Improved DIAAS as a result of protein mixture plant/animal-derived protein.

| **Plant/animal-derived protein mixture** | **Max. DIAAS^1^ (≤100)** | **Ratio** |
| --- | --- | --- |
| Oat/casein | 100 | up to 45% oat |
| Oat/whey | 88 | 60/40 |
| Oat/egg | 100 | up to 10% oat |
| Oat/pork | 94 | up to 55% oat |
| Pea/casein | 100 | up to 35% pea |
| Pea/whey | 93 | 60/40 |
| Pea/egg | 100 | up to 15% pea |
| Pea/pork | 100 | up to 45% pea |
| Corn/casein | 100 | up to 35% corn |
| Corn/whey | 94 | 40/60 |
| Corn/egg | 100 | up to 30% corn |
| Corn/pork | 100 | up to 45% corn |
| Canola/whey | 95 | 60/40 |
| Canola/casein | 100 | up to 55% canola |
| Canola/egg | 100 | up to 10% canola |
| Canola/pork | 100 | up to 50% canola |
| Soy/ casein | 100 | up to 55% soy |
| Soy/ whey | 100 | 45%-70% soy |
| Soy /egg | 100 | up to 55% soy |
| Soy /pork | 100 | up to 70% soy |
| Potato/ casein | 100 | any |
| Potato/ whey | 100 | 100/0 |
| Potato/egg | 100 | any |
| Potato/pork | 100 | any |

^1^DIAAS value derived from average IAA content and average SID per protein sources and calculated according to equations (6), (7) and (8). Based on 0,5-3 years old population reference pattern score.
